# Psittacosis Outbreak in Europe: A Concern for Public Health

**DOI:** 10.1111/crj.70068

**Published:** 2025-03-11

**Authors:** Salomon Izere, Hope Intwari Munyaneza, Faisal Ahmed

**Affiliations:** ^1^ College of Medicine and Health Sciences University of Rwanda Kigali Rwanda; ^2^ Voronezh State Medical University N.N. Burdenko Voronezh Russia; ^3^ Department of Health Sciences and Informatics Bangladesh Institute of Innovative Health Research Dhaka Bangladesh

**Keywords:** avian hosts, *Chlamydophila psittaci*, epidemiological investigations, outbreak, psittacosis, respiratory infection

**Abbreviations**
RT‐PCR: real‐time polymerase chain reaction
WHO: World Health Organization

Dear Editor,

Psittacosis, also known as parrot fever or ornithosis, is a zoonotic bacterial infectious disease caused by *Chlamydia psittaci* , an obligatory intracellular organism [1]. The infection is primarily transmitted through contact with infected avian species, leading to a diverse spectrum of clinical manifestations and severity. *Chlamydia psittaci* predominantly resides in birds, particularly those within the Psittaciformes order, which includes species such as parakeets, parrots, lorikeets, cockatoos, and budgerigars, as well as birds from the Galliformes order, including chickens, turkeys, and pheasants. Notably, any bird species can potentially harbor the disease [2].

The primary risk factor for transmission to humans involves direct contact with infected birds or inhalation of aerosolized pathogens resulting from their urine, feces, respiratory secretions, and ocular exudates (Figure 1) [3, 4, 5]. Although there are occasional reports of human‐to‐human transmission, such occurrences are considered rare. Additionally, humans may contract psittacosis through exposure to *C. psittaci* present in aborted products from equine sources, thus underlining the significance of a One Health approach to understanding the disease.

**FIGURE 1:**
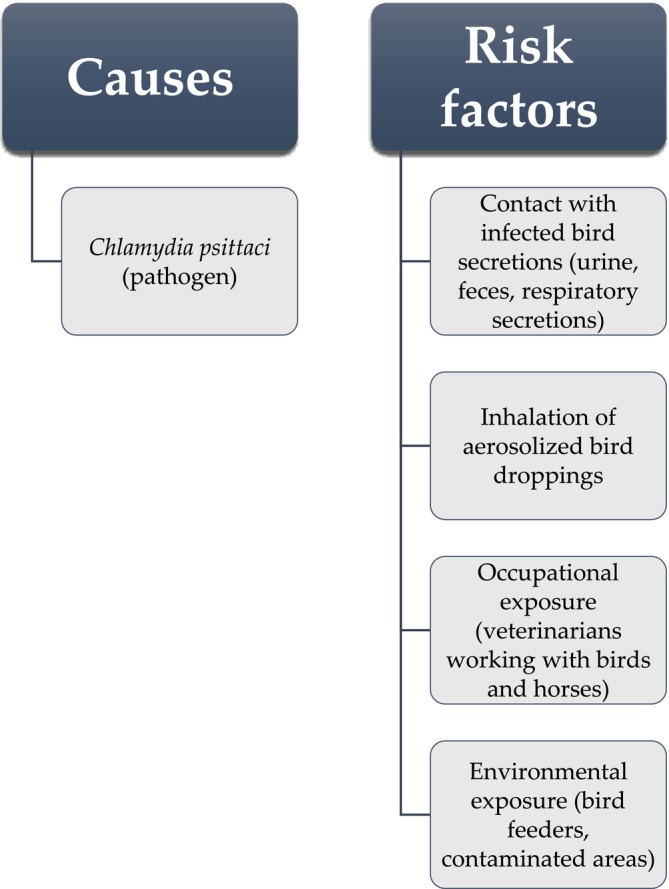
Causes and risk factors of psittacosis.

Symptoms of psittacosis can vary from mild to severe, with complications such as pneumonia occurring frequently and posing a risk of fatality, as evidenced by recent outbreaks documented in Europe [4, 5]. Typically, symptoms manifest within a timeframe of 5 to 14 days following exposure to the pathogen. The management of psittacosis‐related pneumonia necessitates the use of antimicrobial drugs, particularly as pulmonary involvement is prevalent at the time of diagnosis. Currently, antibiotics such as tetracyclines and chloramphenicol are the preferred therapeutic agents. Most patients respond favorably to oral administration of chloramphenicol palmitate, tetracycline hydrochloride, or doxycycline [4]. For critically ill patients, intravenous administration of doxycycline hyclate may be considered an initial treatment option. Symptoms generally begin to remit within a period of 48 to 72 h. It is imperative that, following the resolution of fever, the course of treatment is maintained for a minimum of 10 to 14 days to mitigate the risk of relapse [1, 4].

Psittacosis can affect individuals regardless of age or gender; however, its incidence appears to peak among individuals aged 35 to 55 [4]. The first documented case of psittacosis was identified in 1879 when seven individuals in Switzerland were diagnosed with pneumonia following exposure to tropical pet finches and parrots [6], although the infectious agent was not initially recognized. Subsequent pandemics occurred in 1929 and 1930 [6, 7]. Despite remaining relatively rare, psittacosis is currently regarded as a significant public health concern in various European Countries [8]. Notably, there has been a concerning increase in reported cases in Austria, Denmark, Germany, Sweden, and the Netherlands since late 2023, as detailed in Table 1 [1]. This escalation has prompted epidemiological investigations to identify likely sources of exposure and clusters of the disease. The increase in psittacosis cases during the years 2023 to 2024 may be partially attributed to climatic factors, including changes in temperature, precipitation, or frost patterns, which could affect bird migration, behavior, or the aerosolization of droppings. Further research is warranted to examine these potential influences.

**TABLE 1:** An overview of psittacosis cases in five European countries (2023–2024) [1, 9].

Country	Year	Cases reported	Hospitalizations	Deaths	Exposure details	Severity Details (e.g., pneumonia)	Diagnostic procedures	Data Gaps/Comments
**Austria** [1]	2023	14	Not specified	0	No travel abroad was reported; exposure to birds was not consistently mentioned	Not specified	Serological testing, with lower sensitivity than RT‐PCR, may contribute to underreporting. PCR should be used for better accuracy in future surveillance	Limited info on bird exposure
	2024	4 (as of March)	Not specified	0				
**Denmark** [1]	2023	Increase noted	17	4	Mainly contact with wild birds; some linked to domestic birds	15 cases had pneumonia	Although RT‐PCR was utilized, reliance on less sensitive serological testing in some cases may have resulted in underreporting	Environmental exposure is suspected; data collection ongoing
	2024	23 (as of February)						
**Germany** [1]	2023	14	16	0	26% cases linked to domesticated birds, no info on wild bird exposure for others	18 cases had pneumonia	Antibody/serological testing, which is less sensitive than RT‐PCR, was the primary diagnostic method and may have caused underreporting. RT‐PCR should be prioritized	Missing info on bird exposure
	2024	5 (as of February)						
**Sweden** [1]	2023	26 (Nov–Dec)	Not specified	0	Contact with bird dropping via feeders and some domestic birds	Not specified	Increased RT‐PCR usage has improved detection, but historical reliance on serological testing may have caused underreporting	Changes in diagnostic practices likely contributed to increased detection
	2024	13 (Jan–Feb)	13 (Jan–Feb)					
**Netherland** [1]	2023	21 (Dec–Feb)	All cases hospitalized	1	Mixed exposure: wild and domestic birds; some cases with no bird contact reported	Not specified	RT‐PCR, used for over 95% of notifications, provides greater diagnostic accuracy. However, earlier reliance on less sensitive serological testing may have contributed to underreporting	Wide age range (37–86); geographically spread throughout the country
**Total**	2023–2024	120+	33 + hospitalized	5+	Mixed exposure, primarily wild and domestic birds	High prevalence of pneumonia	Predominantly serological and RT‐PCR Testing	Data gaps in exposure details and diagnostics

As of February 2024, the countries listed in Table 1 reported an alarming rise in psittacosis cases diagnosed both in 2023 and early 2024, particularly from November to December 2023. This surge has resulted in five reported fatalities in these nations, underscoring the seriousness of this public health issue. The majority of the cases involved exposure to either wild or domestic avian species.

A similar trend in a rural Australian town has been noted, where individuals who spend substantial time outdoors or engage in lawn mowing activities exhibit a significantly higher likelihood of contracting psittacosis. This phenomenon may be attributable to the inhalation of aerosolized bird droppings [10].

In Austria, the count of confirmed psittacosis cases in 2023 has exceeded the average number reported over the preceding 8 years, with no instances linked to travel. The absence of wild birds as a recognized source of the illness has left health officials in a state of perplexity. The consistency of diagnostic methods underscores the importance of maintaining vigilance [1, 7, 11]. Conversely, Denmark has experienced a notable uptick in reported cases, with 23 individuals testing positive for *C. psittaci* . The majority of these cases have emerged in northern Denmark, Zealand, and the capital region [12]. Regrettably, four patients were lost to follow‐up. Epidemiological investigations have established connections to wild birds, particularly through the use of bird feeders. Although transmission from chickens has been excluded, the source of infection remains ambiguous. Of the hospitalized individuals, 17 cases (74%) required admission, with 15 suffering from pneumonia, and four fatalities recorded [1].

Over the past 5 years, Denmark has consistently reported between 15 and 30 human cases annually, predominantly associated with exposure to domestic birds, such as parrots, parakeets, and hobby birds like racing pigeons, as well as ducks during hunting activities. However, several cases each year indicate no direct contact with birds, suggesting the possibility of environmental contamination [1, 11]. There is currently no evidence suggesting additional testing or modifications in testing techniques in Denmark that could elucidate the recent increase in psittacosis cases.

As a consequence of inhaling airborne particles from the desiccated droppings of infected birds, the Statens Serum Institute and the National Health Institute of Denmark posit that diseases are predominantly associated with wild birds. To elucidate this matter, samples of wild birds submitted for avian influenza testing will be subjected to examination. Currently, the prevalence of *C. psittaci* among wild birds in Denmark remains unknown [1, 11]. It is surmised that the number of individuals infected with *C. psittaci* exceeds documented cases.

Moreover, in December 2023, Germany reported five additional instances of *C. psittaci* , thereby increasing the total number of confirmed cases to 14 for the year 2023. As of February 20, 2024, five further verified instances of psittacosis have been recorded. Aside from a notable clustering of cases in the Hannover region over the past year, no additional geographic clusters have been reported. Pneumonia affected nearly all cases (18 out of 19), with 16 requiring hospitalization [1, 11]. Of the 19 reported cases from January 1, 2023, to February 19, 2024, only 26% (five out of 19) had associated exposure information related to domesticated birds, such as parrots, chickens, or breeding pigeons. Notably, no cases provided information regarding exposure to wild birds. Germany has maintained an average of 15 cases annually over the last 5 years, peaking at 19 cases in 2022 and reaching a low of 11 in 2019. Typically, one or two cases are reported each month. Antibody testing has confirmed approximately 72% (56 out of 78) of cases recorded in the past 5 years; however, the availability of bird exposure data frequently remains inadequate. In late November and early December 2023, Sweden experienced an unusual increase in psittacosis cases, reporting seven instances in November and 19 in December. This surge indicates a doubling of cases compared to the same months in the previous 5 years [1, 7].

Nevertheless, in January and February 2024, fewer occurrences were reported than during the same period over the preceding five years combined, with 10 cases in January and three in February. Since 2017, there has been a documented overall increase in psittacosis cases in Sweden.

Geographically, eight out of Sweden's 21 regions, concentrated in the southernmost part of the country, have reported documented cases as of early November 2023. A limited number of cases have resulted from contact with droppings of small birds via feeders, while some cases are believed to have originated from domestic birds such as hens or cockatoos. The increase in reported diagnoses may be attributed, in part, to advancements in RT‐PCR testing methodologies. Lastly, The Netherlands has observed a rise in confirmed cases of psittacosis since late December 2023. As of February 29, 2024, 21 individuals have tested positive for *C. psittaci* , double the instances reported during the same time frame the previous year. Over the last decade, the average number of cases during this period has been nine.

There has been a geographic distribution of recent cases across the nation, yet no common source of infection has been identified. The patients' ages ranged from 37 to 86 years, with a predominance of 16 men (76%), on average, representing the older demographic [1, 11]. One recent case resulted in mortality, while the remainder required hospitalization. Since late December 2023, a total of 21 instances have been documented, with six cases associated with contact with wild bird droppings, seven linked to domestic bird droppings, and eight cases occurring without any interaction with birds. The testing protocols in the Netherlands have remained consistent over recent years, which may constitute an additional risk factor contributing to the surge in cases. Since 2018, RT‐PCR testing has accounted for over 95% of reported notifications.

## Conclusion

In response to the current outbreak, we strongly advocate for enhanced surveillance, prompt reporting, and heightened vigilance [13]. It is essential to monitor the prevalence of *C. psittaci* in wild birds and to increase awareness among at‐risk populations as critical public health measures [5]. Although the World Health Organization (WHO) currently assesses the risk as low, collective efforts are required to avert potential tragedies and future outbreaks [14]. Furthermore, it is vital to educate the general public, healthcare professionals, and birdwatchers regarding this pandemic. Increasing the awareness and suspicion of psittacosis is necessary, as the illness can present symptoms similar to other respiratory infections. Implementation of preventive measures, assurance of early diagnosis, and initiation of treatment, typically with doxycycline, are imperative for effective mitigation of the disease's impact. By collaborating in these efforts, we can significantly reduce the consequences of this outbreak, but such endeavors necessitate our immediate attention and cooperation.

## Author Contributions

All authors equally contributed to the preparation of this article.

## Ethics Statement

The authors have nothing to report.

## Conflicts of Interest

The authors declare no conflicts of interest.

## Data Availability

Data sharing not applicable to this article as no datasets were generated or analysed during the current study.
